# Differences in Attitudes Toward Medical Cannabis With Humanized Patient Scenarios

**DOI:** 10.7759/cureus.28354

**Published:** 2022-08-24

**Authors:** Thomas A Clobes, Mya Arellano, Matin Gagnon, Colby Klaiman

**Affiliations:** 1 Health Sciences, California State University Channel Islands, Camarillo, USA; 2 Psychology, California State University Channel Islands, Camarillo, USA; 3 Biology, California State University Channel Islands, Camarillo, USA

**Keywords:** bias, public health, stigma in health care, stigma, medical cannabis, psychoactive drug

## Abstract

Introduction

Attitudes toward medical cannabis are shaped by a number of factors, such as religion, previous use, and political affiliation. Individuals with less supportive beliefs toward cannabis, in general, may be more open to its therapeutic applications in a humanized patient scenario (PS).

Methods

A modified medical cannabis attitude scale was used to measure participants' attitudes toward medical cannabis. Two humanized patient scenarios were presented to the participants, and their level of agreement with the patient having access to medical cannabis was measured. After the scales were standardized, a Wilcoxon signed-rank test (WSR) was utilized to determine whether a difference between medical cannabis attitudes and approval of medical cannabis use in the humanized PS exists.

Results

A total of 645 participants completed the full survey and were included in the data analysis. Most participants were supportive of the patients in the humanized scenarios having access to medical cannabis; 76.1% and 75.7% of respondents, in each of the scenarios, selected the highest level of approval. There was a significantly higher approval for medical cannabis with the PS than attitudes toward medical cannabis in general (Z=-17.415, p<0.0005).

Conclusion

Individuals were more supportive of patient access to medical cannabis when exposed to humanized PS than their general attitudes toward medical cannabis indicated. Applying the results of this current research, a viable plan to reduce the stigma surrounding cannabis is to use patient testimonials in public-facing advocacy efforts regarding medical cannabis.

## Introduction

Cannabis has had a turbulent history in the United States. Attitudes towards cannabis have fluctuated over time, with recent decades seeing a more positive perspective on the plant [1,2]. At the state level, over two-thirds of states have legalized the medical use of cannabis. California started this trend when it legalized cannabis for medicinal use in 1996 [3]. However, cannabis has been labeled a Schedule I drug since the 1971 Controlled Substance Act. This Scheduling of cannabis defines it as both possessing a high potential for abuse while having no approved medical uses [4]. This definition of cannabis under federal law not only greatly impedes research efforts to determine any true therapeutic benefits [5,6], psychoactive or otherwise, but it is possible that it creates feelings of immorality surrounding its use.

Attitudes toward cannabis are shaped by a number of factors, including its legal status, knowledge about the plant, media exposure, and implicit biases from environmental contexts [7]. The personal circumstances surrounding decision-making inherently influence the process. In the case of cannabis, its status as a Schedule I drug influences the decisions people make about it and gives negative undertones to any belief held about its uses. This stigma has the implications of social discrimination as well, with factors such as race and socioeconomic status of the user having an impact on how others view the user [8].

Personal beliefs being in direct conflict with some behavioral choices is not new. This phenomenon is apparent when analyzing past large-scale social movements, such as the HIV/AIDS epidemic [9]. Members of society who typically hold discriminatory views of the lesbian, gay, bisexual, transgender, and queer (LGBTQ) community often contradictorily view close friends and family who identify as LGBTQ with warmth. Even though a person may have held personal beliefs at odds with a friend or family member, an amount of the negativity reduces when real people are factored into the equation. Once awareness of the bias is obtained, it is possible for logical thinking to override previously held beliefs [10]. In the context of medical cannabis, individuals with less supportive beliefs toward cannabis, in general, may be more open to its therapeutic applications in a humanized patient scenario (PS).

A similar concept was investigated by Sznitman and Lewis [11] in a randomized web-based study to determine whether narrative statements increased positive attitudes towards recreational cannabis more than non-narrative information. Researchers found that exposing participants in Israel to testimonials significantly increased positive attitudes and beliefs toward recreational cannabis and did so at a rate greater than that of non-narrative videos. The study, however, only focused on attitudes towards recreational cannabis. Additionally, the political landscape in the United States differs from that of Israel, and the political polarization of cannabis in the United States may have an impact on whether patient narratives are an effective measure for reducing stigma.

The current work sought to expand on the knowledge base by analyzing the impact of humanized patient narratives on attitudes regarding medical cannabis in the United States. The research question guiding this research was: how does a humanized PS change one's attitudes toward medical cannabis? The hypothesis, based on previous research, was that the humanized PS would result in a more favorable view toward medical cannabis.

## Materials and methods

In order to determine if there are differences in attitudes toward medical cannabis with and without humanized PS, a survey was administered to participants. Researchers garnered participation through online social media platforms such as Facebook and used snowball sampling. Additionally, researchers partnered with adult education organizations to reach a larger audience. The study was open to anybody aged eighteen or older willing to participate and that had the technology necessary to complete the web-based survey. The survey was open from February to September 2021.

Scale selection

To accurately determine the view participants had pertaining to cannabis, the Recreational and Medical Cannabis Attitudes Scale (RMCAS) was utilized [12]. This scale, consisting of ten questions, gave participants options to indicate one of five responses ranging from "strongly disagree" to "strongly agree" for each question. The questions were designed to consider several factors that may impact the participant's attitudes towards cannabis. Questions target the impact of current legal status, past use, understood future risk, and beliefs of those closest to them. The scale has been determined to be reliable, with each portion of the survey being analyzed independently. The Medical Cannabis Attitudes Scale (MCAS) had a reported reliability coefficient of 0.86; the Recreational Cannabis Attitudes Scale (RCAS) had a reported reliability coefficient of 0.91 [12].

This current study focused on the medical portion of the RMCAS, modified to remove one question only relevant to older respondents: "When I was 18, I believed that using marijuana for a medical purpose was acceptable". The Modified Medical Cannabis Attitude Scale (MMCAS) consisted of five questions, with a total score ranging from five to 25 after appropriate responses were reverse coded. A previous analysis determined that the removal of one question did not impact the outcome of the scale [13].

Patient scenarios

In addition to the MMCAS, two PS questions were devised to measure real-world beliefs about medicinal cannabis use with a humanized application. PS questions were carefully devised to account for confounding variables. The patients presented in the scenarios had divergent backgrounds and experiences. Participants were again asked to identify their level of agreement with a five-point Likert scale from "strongly disagree" to "strongly agree." The first humanized PS read:

Kristen was diagnosed with post-traumatic stress disorder (PTSD) after returning from her military service. She frequently had nightmares about the events that led to her PTSD and severe insomnia. She was often too tired to work, and this situation started to impact her marriage. Kristen tried medical marijuana on the recommendation of a friend; she has been sleeping better and the nightmares are less frequent and less severe. Do you agree Kristen should have access to medical marijuana for her PTSD?

The second humanized PS was:

After a bad car accident, Jermaine has been dealing with chronic pain that is so debilitating he had to quit working and go on disability. His brain was foggy from the opioid medications he was taking. Jermaine's wife recommended he use medical marijuana to reduce his opioid use. He has done so and is able to focus more with only using marijuana infrequently. He is hoping to be able to return to work. Do you agree Jermaine should have access to medical marijuana for his chronic pain?

Data analysis

SPSS version 27.0 (IBM, Inc., Armonk, USA) was used to complete the data analysis. Statistical significance between medical cannabis attitudes and approval of medical cannabis use in the humanized PS was determined using Wilcoxon signed-rank test (WSR). The sample was determined to be positively skewed in favor of cannabis after viewing Q-Q plots and scatter plots, which eliminated the probability of using a paired samples t-test. The total score for the MMCAS was five to 25. The range for the PS was two to 10. Per Milligan and Cooper [14], the data from the different scales was standardized using the following formula, then converted to the five to 25-point scale: Z=X/(Max X-Min X). All p-values were considered two-tailed, with a statistical significance level set at 0.05.

Ethical considerations

The Institutional Review Board at California State University Channel Islands approved the research protocols (#IO5559). Participants electronically acknowledged the informed consent before participating in the study.

## Results

Participant characteristics

At the closure of the survey, 673 individuals had enrolled in the study. However, 28 respondents were excluded from the analysis due to not completing the entire survey. This resulted in a total of 645 participants.

The participants' demographics are detailed in Table 1. Most participants fell between the ages of 18 and 24 and identified as female, White, non-Hispanic, Christian, moderate politically, educated, working full time, and self-identified as Democratic. Participants who indicated that they had used cannabis for any reason accounted for 72.2% (n=466) of responses, leaving 27.8% (n=179) of participants who had never used cannabis. Out of the 466 participants who had ever used cannabis, 64.6% (n=301) of respondents indicated that they had used cannabis in the last month, and 35.4% (n=165) indicated that they had not. Additionally, of the 301 respondents currently using cannabis, 20.2% (n=94) of participants reported using it for medicinal purposes. Another 23.6% (n=110) of participants reported having used it for medical reasons at any point in their life. Of those participants who had used or are currently using cannabis for medicinal purposes (n=204), 31.4% based their use on the recommendation of a physician or licensed healthcare provider (n=64), and 68.6% of participants had used cannabis without a professional recommendation (n=140; see Table 2).

**Table 1:** Respondents' demographics

Characteristics	N	Percentage
Gender		
Male	154	23.9
Female	489	75.8
Non-binary	2	0.3
Age (years)		
18-24	198	30.7
25-34	113	17.5
35-44	123	19.1
45-54	88	13.6
55-84	74	11.5
65-74	32	5
75-84	14	2.2
85 or older	3	0.5
Hispanic origin		
Hispanic	234	36.3
Non-Hispanic	411	63.7
Race		
White	392	60.8
Black or African American	52	8.1
Asian	42	6.51
American Indian or Alaska Native	4	0.6
Native Hawaiian or Pacific Islander	12	1.9
Other	143	22.2
Highest degree		
Some high school	5	0.8
High school	234	36.3
Trade school	59	9.1
Bachelor's degree	223	34.6
Master's degree	89	13.8
Doctoral degree	35	5.4
Employment status		
Full time	313	48.5
Part time	98	15.2
Unemployed looking for work	31	4.8
Unemployed not looking for work	33	5.1
Retired	73	11.3
Student	77	11.9
Disabled	20	3.1
Political affiliation		
Democratic	325	50.4
Republican	114	17.7
Independent/no party affiliation	141	21.9
Libertarian	21	3.3
Other	14	2.2
Not registered	30	4.7
Political view		
Very liberal	122	18.9
Slightly liberal	161	25
Moderate	257	39.8
Slightly conservative	77	11.9
Very conservative	28	4.3
Religion		
Catholicism/Christianity	384	59.5
Judaism	13	2
Islam	7	1.1
Buddhism	9	1.4
Hinduism	1	0.2
Other religion	54	8.4
No religion	177	27.4

**Table 2:** Respondents’ current and past cannabis use

Cannabis use	N	Percentage
Ever used cannabis		
Yes	466	72.2
No	179	27.8
Ever used cannabis medically		
Yes	110	29.6
No	262	70.4
Medical cannabis used based on physician recommendation		
Yes	64	31.4
No	140	68.6

The participants came from a total of 37 states, with the largest representation being California (73.2%), Missouri (5.9%), Texas (2.5%), and Washington (1.4%). The participants' states of residence were categorized by cannabis legal status: not legal (10.8%), medical only (14.4%), and medical and recreational legal (74.8%).

Cannabis attitudes

Most participants were strongly supportive of the patients in the PS having access to medical cannabis: 76.1% and 75.7% of participants selected "strongly agree" that the patients should have access for PTSD and chronic pain patients, respectively. The mean PS score was 23.03. This is in contrast to a mean MMCAS score of 19.97. There was a total of 518 (77.0%) participants who had a higher approval rating in the PS than in the MMCAS.

Researchers successfully reject the null hypothesis that there is no significant difference between humanized PS approval of medical cannabis use and overall attitude toward medical cannabis. Results from a WSR indicated a significantly higher approval for medical cannabis with the PS than attitudes toward medical cannabis (Z=-17.415, p<0.0005; Table 3). Every category was significant in having a higher PS score than the modified MCAS score (p<0.0005), except for participants who indicated they were disabled and those who listed "other" for their political affiliation (Table 3).

**Table 3:** MMCAS versus PS scores MMCAS - Modified Medical Cannabis Attitude Scale; PS - patient scenario

	N	MMCAS	Patient scenario	Z-value	P-value
Overall	645	19.97	23.03	-17.412	<0.0005
Genders					
Male	154	20.11	23.38	-9.007	<0.0005
Female	489	19.94	22.93	-14.874	<0.0005
Age (years)					
18-24	198	19.35	22.99	-10.08	<0.0005
25-54	113	20.62	23.61	-7.073	<0.0005
55-84	123	20.36	22.99	-7.401	<0.0005
45-54	88	20.01	22.7	-6.688	<0.0005
55-84	74	20.41	22.87	-5.187	<0.0005
65-74	32	19.56	23.36	-4.247	<0.0005
75-84	14	19.5	21.96	-2.668	0.008
Hispanic origin					
Hispanic	234	19.55	22.84	-10.312	<0.0005
Non-Hispanic	411	20.21	23.13	-14.063	<0.0005
Race					<0.0005
White	392	20.32	23.17	-13.392	<0.0005
Black or African American	52	20.08	22.45	-4.339	<0.0005
Asian	42	18.31	22.38	-5.136	<0.0005
Other	143	19.41	23.11	-8.711	<0.0005
Highest degree					<0.0005
High school	234	19.62	23.08	-10.657	<0.0005
Trade school	59	20.29	22.84	-4.812	<0.0005
Bachelor's degree	223	20.4	23.25	-10.144	<0.0005
Master's degree	89	19.75	22.98	-7.173	<0.0005
Doctoral degree	35	19.54	21.71	-3.269	<0.0005
Marital status					
Married	266	19.98	22.85	-11.542	<0.0005
Widowed	21	18.9	21.43	-2.445	0.015
Divorced	62	20.74	23.27	-4.96	<0.0005
Never married	291	19.86	23.22	-11.815	<0.0005
Number of children					
0	318	20.06	23.25	-12.33	<0.0005
1	95	20.03	22.92	-6.133	<0.0005
2	133	20.26	23.31	-8.2	<0.0005
3 or more	99	19.22	22.05	-6.967	<0.0005
Employment status					<0.0005
Full time	313	20.06	22.93	-11.956	<0.0005
Part time	98	19.29	22.86	-7.153	<0.0005
Unemployed looking for work	31	19.55	22.66	-3.702	<0.0005
Unemployed not looking for work	33	21.27	23.26	-3.711	<0.0005
Retired	73	20.73	23.46	-6.258	<0.0005
Student	77	19.43	23.73	-6.992	<0.0005
Disabled	20	19.65	21.25	-1.218	0.223
Political affiliation					
Democratic	325	20.42	23.53	-12.682	<0.0005
Republican	114	18.9	22.06	-7.486	<0.0005
Independent/no party affiliation	141	19.9	22.93	-7.925	<0.0005
Libertarian	21	20.43	23.33	-3.297	<0.0005
Other	14	20.71	22.68	-1.873	0.061
Not registered	30	18.73	21.67	-3.37	0.001
Political view					
Very liberal	122	22.01	24.34	-7.808	<0.0005
Slightly liberal	161	20.29	23.66	-9.334	<0.0005
Moderate	257	19.37	22.49	-10.539	<0.0005
Slightly conservative	77	18.86	22.21	-6.267	<0.0005
Very conservative	28	17.79	20.8	-3.122	0.002
Religion					
Catholicism/Christianity	384	19.45	22.62	-13.25	<0.0005
Other religion	54	20.65	23.33	-4.46	<0.0005
No religion	177	21.07	23.95	-9.862	<0.0005
Ever used cannabis					
Yes	466	20.88	23.76	-15.146	<0.0005
No	179	17.58	21.13	-8.79	<0.0005
Ever used cannabis medically					<0.0005
Yes	110	21.33	24.11	-7.39	<0.0005
No	262	20.15	23.35	-11.718	<0.0005

## Discussion

By comparing the attitudes toward medical cannabis to approval ratings of humanized PS, it is evident that implicit biases inhibit the progress of cannabis as a therapeutic substance. To eliminate these biases and align personal belief with applied practice, the primary factors creating biases must be altered [15]. Previous research has demonstrated that health education on medical cannabis can improve attitudes toward medical uses of the plant [13]. Applying the results of this current research, a viable plan to reduce the stigma surrounding cannabis is to use patient testimonials in public-facing advocacy efforts regarding medical cannabis.

Social factors not directly related to medical cannabis can influence attitudes toward it and reinforce the lingering stigma against its use. For instance, in the context of the opioid crisis, wealth class was a significant factor. Despite both wealthy and low-income individuals using opioids, the wealthier were framed in public dialogue in a much more positive light than the lower-income individuals [16]. Additionally, racial undertones have contributed to conflicting views towards cannabis use. One study compared the media's response to Michael Phelps' use of cannabis in 2009 to two athletes of color. The researchers concluded a more considerate and understanding view of Phelps' use of cannabis was presented in the media relative to the other two athletes [8]. Michael Phelps was viewed in a forgiving manner by the media, while the other two athletes were spoken of negatively despite all three having used cannabis.

The media responses to opioid users and public figures' cannabis use further demonstrate that discrepancies can occur between one's inherent beliefs and practical application of those beliefs. Real-life applications can act to decrease implicit bias towards a substance, such as the humanized PS of medical cannabis users in this study, or it can further exacerbate and amalgamate implicit biases, as in the cases of the two athletes of color mentioned above. Even during a time when medical cannabis legality in the United States is becoming more widespread, social factors such as wealth and race can blur perceptions of cannabis through implicit bias. Also noted in research by Sznitman and Lewis [11], medical cannabis users are more likely accepted if they have an illness that was no fault of their own. Those that have an illness perceived as self-inflicted, such as a substance use disorder, are less likely found to be socially acceptable. Considering the weight that socioeconomic status, race, and pathology of illness have, it is critical that public health initiatives carefully consider the kind of narratives that are employed.

Cannabis as a Schedule I substance presents not only barriers to access for patients but a barrier to information regarding cannabis. The removal of cannabis from Schedule I will improve opportunities to research its therapeutic potential [17]. Additionally, clinical trials investigating the effects of medicinal cannabis could help inform the public of the benefits and risks associated with it. This would only be possible with the loosening of federal cannabis laws [18]. Exploring the effects of medicinal cannabis in a clinical setting will help remove the lingering stigma around the use of cannabis for medical purposes. Even with the status of cannabis as a Schedule I drug and other implicit biases, approval of its use improves when applied to a real-life scenario. As a result, the social barriers preventing patients from seeking medical cannabis as a psychoactive therapeutic option may be easier to overcome than previously understood.

The majority of respondents were registered Democrats. However, given all political party affiliations, excluding those who identified as "other", had a significant difference in humanized PS approval versus MMCAS. Therefore, this disproportionate number of Democrat respondents likely had little impact on the final results. Further, Democrats had the second highest MMCAS, and there was still a significantly higher approval when the PS was provided.

One strength of this study is the practical application of health education efforts to reduce stigmas toward improving patient access to medical cannabis. While health education can reduce stigma toward medical cannabis, including relevant patient testimonials in the education efforts is a manageable task to improve its effectiveness [13]. This study also used patient scenarios that account for most of the medical cannabis use: chronic pain and PTSD [13,19,20].

One limitation of this study, though, is the lack of measuring the direct impact of patient-focused testimonials on attitudes toward medical cannabis. In previous research, the approval ratings for medical cannabis access with specific patients improved with nonpatient specific education [13]. Measuring attitudes toward medical cannabis before and after patient-focused education is a logical next step. Another limitation is the high number of respondents who reside in states with full access to cannabis; repeating this study with a higher proportion of respondents from states without any access to cannabis or medical-only access may yield different results.

## Conclusions

Numerous factors influence one's attitudes toward medical cannabis. The lingering stigma toward medical cannabis limits patients' access to the therapeutic benefits of the plant. There is a discrepancy between individuals' attitudes toward medical cannabis when it is and when it is not directly linked to a humanized patient story. Including patient testimonials in medical cannabis advocacy efforts will help improve patient access.

## References

[1] Felson J, Adamczyk A, Thomas C (2019). How and why have attitudes about cannabis legalization changed so much?. Soc Sci Res.

[2] Carliner H, Brown QL, Sarvet AL, Hasin DS (2017). Cannabis use, attitudes, and legal status in the U.S.: a review. Prev Med.

[3] Vitiello M (1998). Proposition 215: de facto legalization of pot and the shortcomings of direct democracy. U Mich JL Reform.

[4] Siff S (2014). The illegilization of marijuana: a brief history. https://origins.osu.edu/article/illegalization-marijuana-brief-history?language_content_entity=en.

[5] Satterlund TD, Lee JP, Moore RS (2015). Stigma among California's medical marijuana patients. J Psychoactive Drugs.

[6] Luque JS, Okere AN, Reyes-Ortiz CA, Williams PM (2021). Mixed methods study of the potential therapeutic benefits from medical cannabis for patients in Florida. Complement Ther Med.

[7] Dietrich C (2010). Decision making: Factors that influence decision making, heuristics used, and decision outcomes. Inq J.

[8] Lewis J, Proffitt JM (2012). Bong hits and water bottles: an analysis of news coverage of athletes and marijuana use. J Sports Media.

[9] Vanlandingham MJ, Im-Em W, Saengtienchai C (2005). Community reaction to persons with HIV/AIDS and their parents: an analysis of recent evidence from Thailand. J Health Soc Behav.

[10] West RF, Toplak ME, Stanovich KE (2008). Heuristics and biases as measures of critical thinking: Associations with cognitive ability and thinking dispositions. J Educ Psychol.

[11] Sznitman SR, Lewis N (2018). Examining effects of medical cannabis narratives on beliefs, attitudes, and intentions related to recreational cannabis: A web-based randomized experiment. Drug Alcohol Depend.

[12] Arora K, Qualls SH, Bobitt J, Lum HD, Milavetz G, Croker J, Kaskie B (2020). Measuring attitudes toward medical and recreational cannabis among older adults in Colorado. Gerontologist.

[13] Clobes TA, Palmier LA, Gagnon M, Klaiman C, Arellano M (2022). The impact of education on attitudes toward medical cannabis. PEC Innovation.

[14] Milligan GW, Cooper MC (1988). A study of standardization of variables in cluster analysis. J Classif.

[15] Lashley K, Pollock TG (2020). Waiting to inhale: Reducing stigma in the medical cannabis industry. Adm Sci Q.

[16] Tiger R (2017). Race, class, and the framing of drug epidemics. Contexts.

[17] Andreae MH, Rhodes E, Bourgoise T (2016). An ethical exploration of barriers to research on controlled drugs. Am J Bioeth.

[18] Hutchison KE, Bidwell LC, Ellingson JM, Bryan AD (2019). Cannabis and health research: rapid progress requires innovative research designs. Value Health.

[19] Walsh Z, Callaway R, Belle-Isle L, Capler R, Kay R, Lucas P, Holtzman S (2013). Cannabis for therapeutic purposes: patient characteristics, access, and reasons for use. Int J Drug Policy.

[20] Haug NA, Padula CB, Sottile JE, Vandrey R, Heinz AJ, Bonn-Miller MO (2017). Cannabis use patterns and motives: A comparison of younger, middle-aged, and older medical cannabis dispensary patients. Addict Behav.

